# Poly(hexamethylene guanidine): An Effective Compound in Tackling Persistent Bacterial Subpopulations

**DOI:** 10.3390/microorganisms13092002

**Published:** 2025-08-27

**Authors:** Weilin Liu, Jiang Zhang, Liang Chen

**Affiliations:** 1 College of Bioengineering, Beijing Polytechnic University, Beijing 100176, China; liuweilin@bpi.edu.cn; 2 College of Life Science and Technology, Beijing University of Chemical Technology, Beijing 100029, China; 19109643649@163.com

**Keywords:** persistent bacteria, antibiotic resistance, cationic polymers, poly(hexamethylene guanidine), antibacterial performance

## Abstract

Persistent bacteria (PB) are a subpopulation of dormant cells that tolerate high antibiotic concentrations and cause chronic, hard-to-treat infections, posing a serious global health threat. In this study, the antibacterial efficacy of six cationic polymers, poly(hexamethylene guanidine) (PHMG), polyethyleneimines of different molecular weights, α-polylysine, ε-polylysine, and polyacrylamide, against persistent bacteria was systematically evaluated. The minimum inhibitory concentration (MIC) and minimum bactericidal concentration (MBC) of these cationic polymers against susceptible and persistent methicillin-susceptible *Staphylococcus aureus* (MSSA), methicillin-resistant *S. aureus* (MRSA), and *Escherichia coli* (*E. coli*) were determined using a microbroth dilution method, while cytotoxicity to mouse fibroblast (L929) cells was assessed via MTT assay. PHMG demonstrated superior antibacterial activity, with MBC values as low as 2 μg/mL against persistent MSSA, markedly outperforming the other polymers tested. The key novelties of this work are (i) the first establishment of a cationic polymer library with diverse structural parameters for persistent bacteria clearance, offering a potential strategy for treating recalcitrant infections; and (ii) the elucidation of quantitative correlations between polymer charge density and hydrophobic chain segments with antimicrobial efficacy through structure–activity relationship analysis, providing a theoretical basis for the rational design of anti-persistent materials.

## 1. Introduction

Bacterial infections, a global challenge threatening human health, have been widely recognized as clinical hazards [1,2]. Bacterial infections have become the second leading cause of death after ischemic heart disease, with approximately one in eight deaths worldwide in 2019 associated with bacterial infections [3]. Although the immune system can clear some invading pathogens, effective interventions are still essential during infection outbreaks [4]. While clinical use of antibiotics has saved countless lives, overuse has led to the proliferation of multidrug-resistant strains, with resistance mechanisms such as drug efflux, enzymatic inactivation, and target modification thoroughly analyzed [3,5,6,7]. Notably, some bacteria evade antibiotic killing by entering a metabolic dormant state, and this phenotypically resistant subpopulation, defined as persistent bacteria, represents a major barrier to eradicating infections [8,9,10].

The biological characteristics of persistent bacteria, first identified by Bigger in 1944 in penicillin-treated *Staphylococcus aureus* (*S. aureus*) [11], are of significant clinical importance [12,13]. Persister bacteria refer to a subpopulation of cells that can survive in the presence of antibiotics but do not possess genetic resistance [8]. They are characterized by viability in lethal antibiotic concentrations (often exceeding 10-fold the MIC) [8,14], comprising <0.1% of the population [9,15], non-replicating or low-metabolic states [16], and phenotype-based resistance independent of genetic mutations [13], which is reversible [17]. The resistance mechanism of persister bacteria mainly depends on reversible regulation of physiological state rather than genetic mutation or acquisition of resistance genes, making it fundamentally different from antibiotic-resistant bacteria [9]. In this state, essential cellular processes, including DNA replication, protein synthesis, energy metabolism, and cell wall biosynthesis, are markedly downregulated or temporarily arrested [6,15,18]. Since most bactericidal antibiotics require active metabolism to exert their effects (e.g., *β*-lactams targeting cell wall synthesis and aminoglycosides depending on protein translation), these drugs become ineffective against metabolically inactive cells. As a result, persisters exhibit transient survival in the presence of otherwise lethal antibiotic doses [19]. Additionally, some persister cells further suppress metabolic activity and enhance stress tolerance by activating toxin–antitoxin modules, modulating intracellular ATP levels, and strengthening oxidative stress defenses [20]. Notably, this form of tolerance is phenotypic and non-genetic; persisters can resume normal growth once antibiotic pressure is removed, contributing to the clinical challenge of chronic and recurrent infections [21,22]. Biofilms are considered the primary niches where persistent bacteria reside. Bacteria within biofilms exist in a microenvironment characterized by hypoxia, limited energy uptake, and restricted metabolism, which triggers responses from the SOS system, as well as the (pp)pGpp and toxin–antitoxin (TA) systems, thereby facilitating bacterial entry into the persistent state [16]. Consequently, bacterial biofilms are believed to be associated with 80% of refractory bacterial infections in clinical settings [21,23].

Current strategies to eliminate persistent bacteria focus on three directions: direct disruption of biofilm and cell membrane structures (e.g., lysozyme, antimicrobial peptide mimetics) [16]; induction of dormant cell resuscitation to restore antibiotic susceptibility (e.g., metabolic activator cis-2-decanoic acid) [24,25,26]; and inhibition of persistence formation mechanisms (e.g., interference with quorum sensing systems) [27,28]. Among these, physical disruption strategies targeting the cell membrane have gained much attention due to their independence from bacterial metabolic states [29,30]. Cationic polymers, electrostatically adsorbed onto the negatively charged bacterial membrane surface, insert hydrophobic chain segments into the phospholipid bilayer, leading to membrane integrity loss—a mechanism circumventing the resistance evolution pathway of conventional antibiotics [31,32]. Studies have shown that cationic polymers like methacrylate copolymers containing amine/quaternary ammonium groups efficiently kill planktonic bacteria with controllable hemolytic toxicity, offering new insights for anti-persistence research [33,34]. Cationic dendrimers (e.g., PAMAM and peptide dendrimers) with a tree-like structure and high multivalence exert antibacterial activity by disrupting bacterial membranes via electrostatic interactions and hydrophobic insertion, and some can penetrate extracellular polymeric substances to exhibit antibiofilm effects [35,36]. Quaternary phosphonium salts, a novel class of cationic compounds, show superior stability and antibiofilm activity compared to quaternary ammonium salts [37], offering new insights for anti-persistence research.

**Scheme 1:**
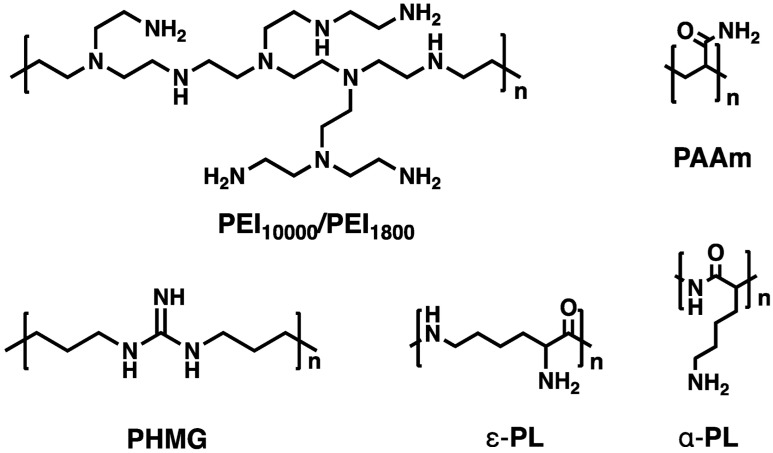
Structures of cationic polymers.

Against this backdrop, this study systematically evaluated the efficacy of cationic polymers in eliminating persistent bacteria. Six cationic polymers, including poly(hexamethylene guanidine) (PHMG), polyethyleneimines (PEIs) with different molecular weights (1800, namely PEI_1800_; 10,000, namely PEI_10000_), α-polylysine (α-PL), ε-polylysine (ε-PL), and polyacrylamide (PAAm) were screened for eliminating persistent bacteria (Scheme 1). The minimum inhibitory concentration (MIC) and minimum bactericidal concentration (MBC) of the cationic polymers against methicillin-susceptible *S. aureus* (MSSA), methicillin-resistant *S. aureus* (MRSA), and *Escherichia coli* (*E. coli*) and their persistent subpopulations were determined. Study novelties include firstly, the establishment of a cationic polymer library encompassing PHMG, PEIs, α-PL, and ε-PL, providing a paradigm for screening materials for clinical persistent bacterial infection interventions; secondly, the analysis of polymer charge characteristics (e.g., guanidinium group complete protonation at physiological pH) and hydrophobic membrane perturbation capabilities (e.g., amphiphilic structure penetration into the phospholipid bilayer) to establish a structure–activity relationship model, offering theoretical support for designing antimicrobial materials to disrupt persistent bacterial membrane structures.

## 2. Experimental Section

### 2.1. Materials

Tryptone Soy Agar (TSA), Tryptone Soy Broth (TSB), Mueller–Hinton Broth (MHB,), and 5 × M9 Basic Salt were purchased from Sinopharm Chemical Reagent Co., Ltd. (Shanghai, China). Vancomycin (≥98%) was obtained from Beijing Bailingwei Chemical Technology Co., Ltd. (Beijing, China). Cationic polymers with different molecular weights, including PHMG (*M*_W_ 533, 99%), PEIs (*M*_W_ 1800 and 10,000, 99%), ε-PL (*M*_W_ 3600–4300, 95%), α-PL (*M*_W_ 3000–5000, 95%), and PAAm (*M*_W_ 300,000, 95%), were supplied by Shanghai McLean Biochemical Technology (Shanghai, China).

### 2.2. Construction of Persistent Bacterial Model

MSSA (ATCC 25923), *E. coli* (ATCC 25922), and MRSA (1857) were used as experimental strains. MRSA 1857 were isolated from the Affiliated Hospital of the Chinese Academy of Military Medical Sciences [38]. After recovery, the bacteria were streaked onto TSA plates and incubated at 37 °C for 24 h. Single colonies were then inoculated into 100 mL of TSB and cultured at 37 °C with shaking at 200 rpm for 24 h. Subsequently, 100 μL of the bacterial culture was transferred into 100 mL of MHB and incubated under the same conditions for 12 h. To induce the persistent bacterial model, 40 mL of the resulting bacterial suspension was mixed with 200 μL of vancomycin solution (10 mg/mL; final concentration) and incubated at 37 °C with shaking at 200 rpm for 8 h [8,39]. The obtained persisters were washed three times by M9 medium and resuspended in M9. The suspension was standardized to 10^6^ CFU/mL. To eliminate variations in the states of persistent bacteria induced by different antibiotics, vancomycin was also used to induce persistent *E. coli* [40,41].

### 2.3. Antibacterial Activity Evaluation

The minimum inhibitory concentrations (MICs) and minimum bactericidal concentrations (MBCs) of the cationic polymers were determined using the microdilution method. For bacterial suspension preparation, 8 mL of bacteria in the exponential phase was divided into four centrifuge tubes and centrifuged at 7500 rpm for 3 min. The supernatant was discarded, and the bacterial pellet was resuspended in 2 mL of saline. This washing step was repeated three times. The final bacterial suspension was adjusted to 2 mL with saline. After gradient dilution and plate counting to determine bacterial concentration, the suspension was standardized to 10^6^ CFU/mL.

Given that assessments involving persistent cells must be conducted in M9 solution, MIC and MBC assays for sensitive bacteria are also performed in M9 medium to ensure comparability. Cationic polymer solutions were prepared by dissolving the compounds in either saline or M9 medium and diluting them to a stock concentration of 2048 μg/mL. For MIC determination, 100 μL of serially diluted polymer solutions (ranging from 1024 to 2 μg/mL) and 100 μL of bacterial suspension were added to 96-well plates. Positive controls consisted of 100 μL of saline mixed with 100 μL of bacterial suspension, and negative controls consisted of 200 μL of medium alone. Each condition was tested in triplicate. The plates were incubated at 37 °C for 24 h, and bacterial growth was assessed by measuring absorbance at 600 nm using a microplate reader. The MIC was defined as the lowest concentration of the polymer that completely inhibited visible bacterial growth.

To determine the MBC, 100 μL of bacterial suspension was taken from the wells showing no visible growth in the MIC assay and spread onto TSA plates, or 10 μL was spotted for drop-plating. The plates were then incubated at 37 °C for 24 h. The MBC was recorded as the lowest concentration at which no bacterial colonies were observed. The assay for persisters was conducted using an agar-based method. An amount of 100 μL of the planktonic persister suspension and cationic polymers at different concentrations were inoculated into 100 mL of M9 medium. The cultures were incubated at 37 °C with shaking at 180 rpm. After 24 h, samples were collected, washed three times with saline, serially diluted, and drop-plated onto TSA plates. Colony formation was recorded after incubation to determine the bacterial survival.

The bacterial growth on TSA medium plates was photographed and documented, with a green-colored background sheet placed underneath during photography to enhance the visibility of the bacteria in the images.

### 2.4. Cytotoxicity Test

The MTT assay evaluated drug cytotoxicity on mouse fibroblast L929 cells. Cells were adjusted to 8000–10,000 cells/well, inoculated into 96-well plates (100 μL/well), and cultured at 37 °C for 24 h. After incubation, the medium was removed, and 100 μL of drug solutions at different concentrations were added to the wells. The cells were further incubated for 24 h, with six replicates for each concentration. Subsequently, 10 μL of MTT solution (5 mg/mL) was added to each well and incubated for 4 h. The supernatant was then carefully removed, and 100 μL of SDS solution was added to dissolve the formed formazan crystals. The plates were kept overnight in the dark. The following day, absorbance was measured at 570 nm using a microplate reader. Cell viability was calculated, and drug cytotoxicity was assessed based on the relative absorbance values.

## 3. Results

### 3.1. Basic Characteristics of Cationic Polymers

As shown in Scheme 1, five classes of six cationic polymers, including PEIs (PEI_10000_, *M_W_* 10,000; PEI_1800_, *M_W_* 1800), PHMG (*M_W_* 533), ε-PL (*M_W_* 3600–4300), α- PL (*M_W_* 3000–5000), and PAAm (*M_W_* 300,000), were selected to study their killing effects on sensitive, resistant, and persistent bacteria. Their structures were confirmed by ^1^H NMR (Figures S1–S5). These cationic polymers are widely used in antimicrobial materials and drug delivery systems due to their excellent antimicrobial properties [30,34,42]. Although all rely on electrostatic interactions between material surface positive charges and bacterial membrane negative charges, they differ in structural composition, cationic group types, biocompatibility, and bactericidal mechanisms.

PEI, a polymer with amine groups, effectively adsorbs and disrupts bacterial membranes via strong cationicity, causing osmotic imbalance and content leakage [43]. However, the strong cytotoxicity of high-molecular-weight PEI limits its biomedical applications [44]. PHMG, with guanidine as the cationic group, exhibits stronger electrostatic adsorption and membrane penetration, destroying membrane structure and penetrating cells, showing excellent bactericidal activity against intracellular bacteria, widely used in disinfectants and medical antibacterial materials [34,45]. Natural peptide cationic polymers like ε-PL and α-PL offer far better biocompatibility than synthetic polymers due to their natural origin and biodegradability, primarily relying on amino groups for electrostatic interactions with bacterial membranes to destroy structure, while PL also induces increased intracellular reactive oxygen species (ROS) to enhance bactericidal effects [46,47]. Unlike these, PAAm lacks inherent antimicrobial properties but is often used as a hydrophilic monomer in hydrogel or functional material matrices, endowed with antimicrobial activity by doping with cationic polymers or agents [48].

### 3.2. Antimicrobial Properties of Cationic Polymers Against MSSA

**Figure 1:**
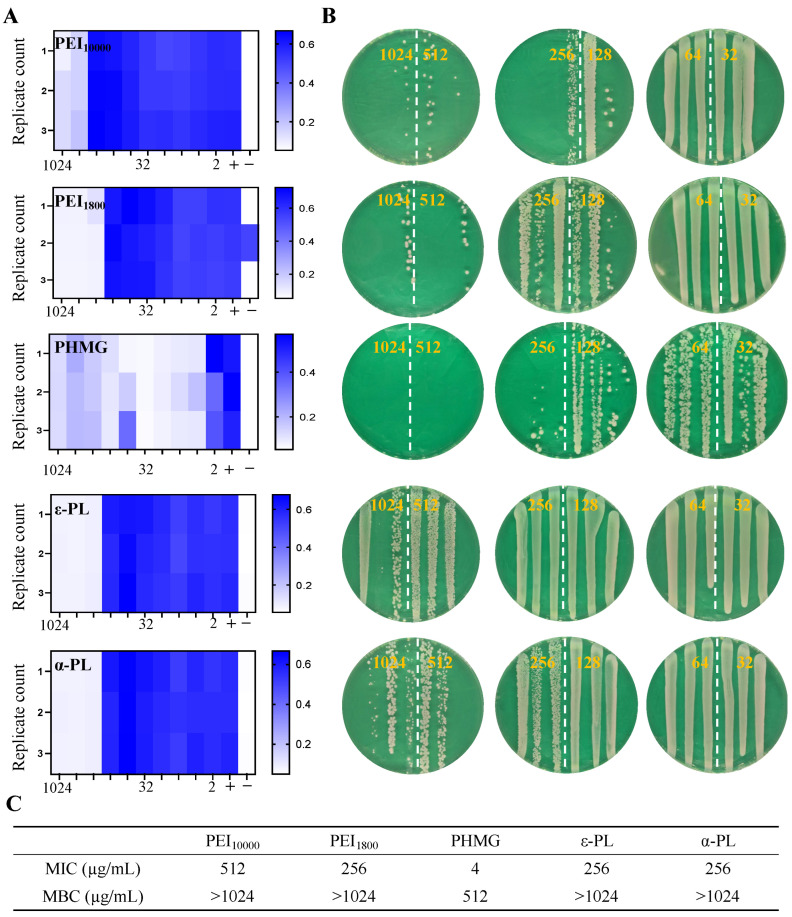
Antimicrobial activity of cationic polymers against MSSA. (**A**) MIC and (**B**) MBC determination of cationic polymers against MSSA. The unit of polymeric concentration is µg/mL. (**C**) Summary of MIC and MBC values.

First, MIC and MBC of the cationic polymers against MSSA were evaluated. As shown in Figure 1A, we used a heatmap to visualize the antibacterial efficacy of cationic polymers. Darker blue indicates a higher bacterial count, while lighter blue corresponds to a lower bacterial count. At a certain concentration, when there was a significant transition from blue to light blue or white in the heatmap, we considered this concentration as the MIC. PAAm exhibited no inhibitory activity at concentrations below 1024 μg/mL (Figure S6). Similarly, chitosan was evaluated as a control. MIC assessment results (Figure S7) showed that even at a concentration as high as 1024 µg/mL, chitosan displayed no antibacterial activity, with bacterial concentrations comparable to those in the nondrug-treated group. This inactivity is likely due to their lack of cationic properties and protonatable amine/guanidine groups, so they were excluded from subsequent studies. When compared to PAAm and chitosan, which lacks antimicrobial activity, other cationic polymers demonstrated significant bacteriostatic effects (Figure 1A). PEI_10000_ and PEI_1800_ had MICs of 512 μg/mL and 256 μg/mL, respectively. Their cationic moieties, originating from primary, secondary, and tertiary amines on main/branched chains, undergo protonation at pH 5.0–7.4, facilitating electrostatic interactions with bacterial membranes and subsequent disruption [49]. Low-molecular-weight PEI_1800_ exhibited a higher cationic group density than PEI_10000_ at the same mass, conferring superior bactericidal properties. The natural peptide-based cationic polymers ε-PL and α-PL displayed identical bacteriostatic effects (MIC 256 μg/mL), as both comprise lysine residues with cationic moieties derived from ε- or α-amino groups [50,51]. Their cationic density is governed by the degree of polymerization and lysine content; similar structural motifs and molecular weights thus resulted in comparable antimicrobial activities. In contrast, PHMG showed the most significant inhibition (MIC 4 μg/mL), far lower than other polymers. The guanidinium cationic moiety of PHMG, fully protonated at physiological pH, establishes a stable, high-density positive charge center through nitrogen interatomic resonance [52]. This characteristic contrasts with amine groups, where protonated charges are localized on a single nitrogen atom, resulting in lower charge density and stability [53]. Additionally, the exceptional hydrogen bond donor capacity of guanidinium enables multi-site coordination with carboxyl and phosphate groups on bacterial membranes, enhancing adsorption stability [34,54]. The cell-penetrating peptide-like properties of the guanidinium group facilitate the formation of transient ion pairs with membrane anionic components, synergistically promoting transmembrane delivery of the polymer [45]. The amphiphilic architecture of PHMG, comprising hydrophobic methylene chain segments and polar guanidinium groups, facilitates insertion into the lipid bilayer, synergistically disrupting membrane structure or forming nanopores, thereby significantly enhancing bactericidal efficacy.

MBC evaluation (Figure 1B,C) showed bactericidal performance of these cationic polymers trended with MIC results. PHMG had the best bactericidal performance (MBC 512 μg/mL), while PEI_10000_, PEI_1800_, ε-PL, and α-PL lacked bactericidal activity below 1024 μg/mL, indicating they could inhibit but not kill bacteria. The bactericidal effect of the cationic polymers on MSSA was further investigated using scanning electron microscopy (Figure S8). As can be seen from the figure, PHMG exerted a significant bactericidal effect. A greater number of MSSA had lost their normal morphological features, while some exhibited clear signs of cell membrane damage, which were characterized by the presence of perforations. This observation directly validates the potent membrane-disrupting capability of PHMG. A similar phenomenon was observed in both the PEI and α-PL groups, albeit significantly less pronounced than in the PHMG group. The underlying mechanism may involve incomplete disruption of bacterial cell integrity [52]. During electrostatic adsorption, if molecular configuration, such as the flexible branched chains of PEI or the hydrophilic nature of ε-PL, results in insufficient hydrophobicity or excessive steric hindrance, these polymers primarily neutralize membrane charges and transiently interfere with metabolic processes (e.g., nucleic acid function or energy homeostasis) without effectively penetrating or disintegrating the phospholipid bilayer [55,56]. This temporary suppression enables bacteria to repair damage and resume activity once the stress is removed [57]. In contrast, polymers like PHMG, which combine high charge density with a rigid hydrophobic structure, induce irreversible membrane tearing, triggering cytoplasmic leakage and ensuring complete microbial eradication.

### 3.3. Antimicrobial Performance of Cationic Polymers Against MRSA

**Figure 2:**
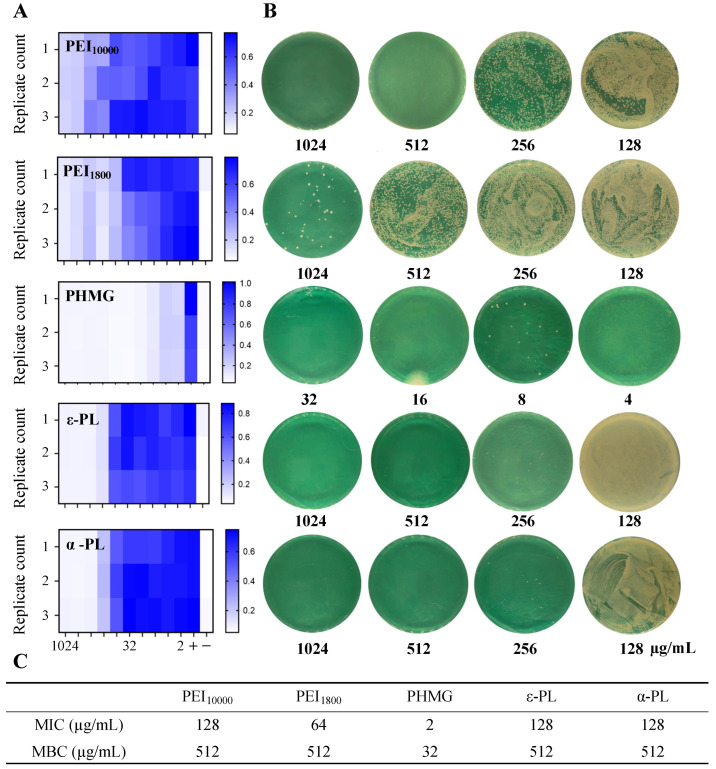
Antimicrobial activity of cationic polymers against MRSA. (**A**) MIC and (**B**) MBC determination of cationic polymers against MRSA; the unit of polymeric concentration is µg/mL. (**C**) Summary of MIC and MBC values.

Subsequently, bactericidal properties against MRSA were evaluated (Figure 2). The essential difference between MRSA and susceptible MSSA lies in its unique resistance mechanism. MSSA may resist penicillin antibiotics via β-lactamase production (overcome by β-lactamase inhibitors), while MRSA expresses a special penicillin-binding protein, PBP2a, due to carrying the mecA gene (or variant mecC) [58]. PBP2a has extremely low affinity for β-lactam antibiotics, enabling continuous cell wall synthesis in drug presence, leading to inherent broad-spectrum resistance to all β-lactams (including methicillin, cephalosporins, etc.) [59]. Mediated by the mobile genetic element SCCmec gene cassette, MRSA often accompanies multidrug resistance and is sensitive only to a few antibiotics like vancomycin [58]. Although MRSA exhibits broad resistance to β-lactams, this does not compromise the bactericidal efficacy of cationic polymers. In fact, these polymers generally demonstrate enhanced antimicrobial activity against MRSA compared to MSSA. MIC data (Figure 2A) revealed that PEI_10000_ and PEI_1800_ achieved MIC values of 128 μg/mL and 64 μg/mL, respectively. ε-PL and α-PL exhibited significantly reduced MICs of 128 μg/mL when compared with those against MSSA. Consistently, PHMG displayed superior bactericidal potency (MIC 2 μg/mL). Bactericidal activity assays further indicated that PEI_10000_, ε-PL, and α-PL had MBCs of 512 μg/mL, whereas PHMG achieved an MBC of 32 μg/mL (Figure 2B,C). These findings underscore the ability of cationic polymers to circumvent bacterial resistance mechanisms, primarily because their mode of action targets the cell membrane, which is unaffected by PBP2a mutation in MRSA [59]. Additionally, to resist cell wall synthesis inhibitors such as β-lactams, MRSA might develop a thicker cell wall compared to sensitive strains (MSSAs). As observed via TEM by Ana Belén García et al. [60], the average cell wall thickness of MRSA (24.42 ± 4.64 nm) is significantly greater than that of MSSA (17.20 ± 2.58 nm), with this difference being statistically significant (*p* < 0.001). A thicker cell wall may confer a more negatively charged surface to MRSA [61]. This increased charge density enhances the electrostatic targeting of MRSA by cationic polymers [62,63]. Moreover, under the pressure of adaptive resistance, cell membranes are often in an abnormal state (e.g., abnormal lipid composition, altered membrane potential), rendering them susceptible to exogenous membrane disruptors such as cationic polymers [30]. Consequently, cationic polymers exhibit higher efficacy against MRSA.

### 3.4. Antimicrobial Performance of Cationic Polymers Against E. coli

Based on the above results, cationic polymers were further evaluated for their inhibitory and bactericidal effects against Gram-negative bacteria (*E. coli*). The primary difference between Gram-positive and Gram-negative bacteria lies in their cell wall structures. Gram-positive bacteria have a thick peptidoglycan layer without an outer membrane, whereas Gram-negative bacteria consist of a thin peptidoglycan layer and an outer membrane composed of lipopolysaccharide (LPS) [64]. This structural disparity not only affects cellular responses to dyes and molecular permeability but also determines differences in sensitivity to antimicrobial agents and exogenous cationic polymers [45].

**Figure 3:**
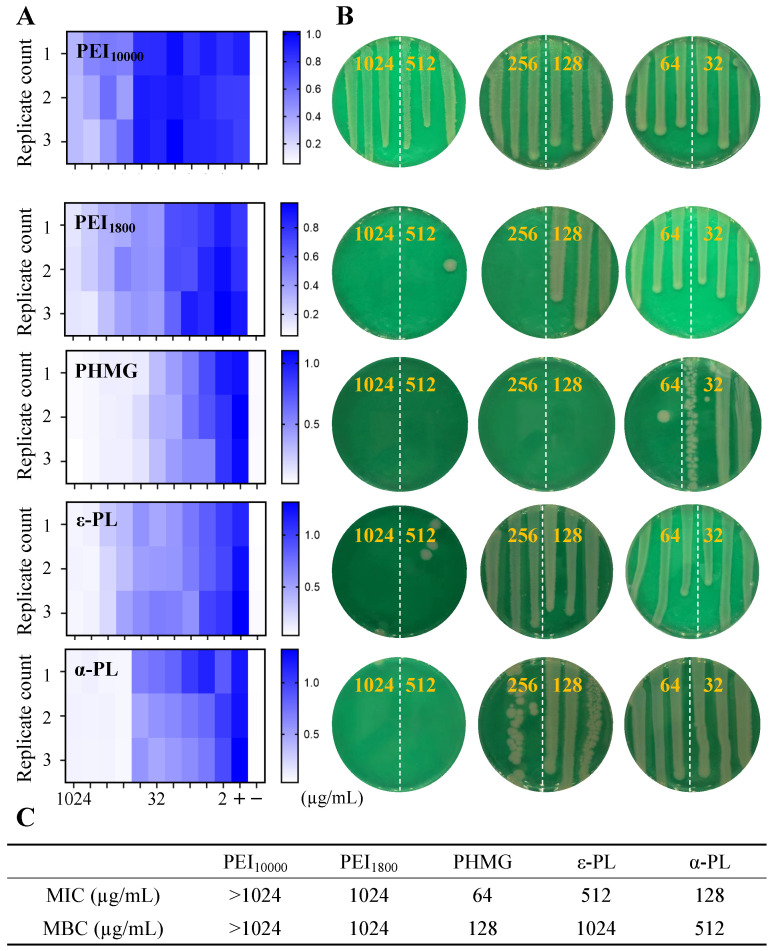
Antimicrobial activity of cationic polymers against *E. coli*. (**A**) MIC and (**B**) MBC determination of cationic polymers against *E. coli*; the unit of polymeric concentration is µg/mL. (**C**) Summary of MIC and MBC values.

Antimicrobial performance evaluation (Figure 3) showed that PEI_10000_ exhibited no inhibitory or bactericidal activity even at a concentration of 1024 μg/mL, while low-molecular-weight PEI_1800_ had both MIC and MBC values of 1024 μg/mL (Figure 3A–C). ε-PL and α-PL demonstrated stronger inhibitory and bactericidal activities against *E. coli*. ε-PL had an MIC of 512 μg/mL and an MBC of 1024 μg/mL, while α-PL showed an MIC of 128 μg/mL and an MBC of 512 μg/mL. α-PL exhibited higher bactericidal activity against *E. coli* than PEI, primarily attributed to its superior biocompatibility and stronger membrane affinity due to its natural amino acid composition. The ε-amino groups on the PL molecular chain are highly protonated under physiological conditions, enabling stronger electrostatic interactions and hydrophobic insertion with the LPS and phospholipid bilayer on the surface of *E. coli*, rapidly disrupting membrane structure and leading to leakage of intracellular components [65]. Although PEIs contains numerous cationic groups, its branched structure and high molecular rigidity limit their uniform distribution and deep perturbation on the bacterial membrane [66], resulting in relatively weak bactericidal effects. Consistent with the above results, PHMG showed the strongest antibacterial activity against *E. coli*, with MIC and MBC values of 64 μg/mL and 128 μg/mL, respectively. PHMG showed lower bactericidal activity against *E. coli* compared to MSSA, which was primarily attributed to differences in cell wall structure between these two bacterial types. *E. coli* possesses an outer membrane composed of LPS, which could effectively hinder the penetration of PHMG and thereby reduce its bactericidal rate and efficiency. Consequently, PHMG demonstrated weaker membrane-disrupting ability against Gram-negative bacteria, resulting in relatively lower bactericidal efficacy.

The bactericidal activity of PHMG against *E. coli* was higher than that against MSSA, mainly due to differences in cell wall structure and surface charge characteristics between the two. As a Gram-negative bacterium, *E. coli* has a large number of negative charges in the LPS layer of the outer membrane [67], which readily undergoes strong electrostatic adsorption with the guanidine groups on PHMG molecules, promoting their penetration into the outer membrane and phospholipid bilayer, rapidly destroying the membrane structure and leading to leakage of intracellular components. In contrast, MSSA is a Gram-positive bacterium that, although lacking an outer membrane, has a structurally dense thick peptidoglycan layer with a limited number of negatively charged sites, which partially hinders the adsorption and penetration of PHMG molecules, reducing its bactericidal rate and efficiency [40,52]. Therefore, PHMG showed stronger membrane disruption ability and higher bactericidal efficacy when acting on Gram-negative bacteria.

### 3.5. Antimicrobial Performance of Cationic Polymers Against Persistent MSSA

Persistent bacteria were constructed with reference to the literature methods [39,68,69]. A high dose of vancomycin (50 × MIC) was added to bacterial suspensions in the stationary phase for 8 hours of co-incubation. During this time, sensitive bacteria were killed by vancomycin, and persistent bacterial subpopulation resistant to antibiotics were screened. The ability of persister bacteria to tolerate high concentrations of antibiotics primarily depends on their reversible transition into a dormant or hypoactive metabolic state [70]. Accordingly, vancomycin was reused to validate the successful construction of the persister model. At concentrations of 10 × MIC and 50 × MIC, it was applied to eliminate persisters. As shown in Figure S9, vancomycin exhibited almost no ability to clear persister cells, confirming that the model was successfully established.

**Figure 4:**
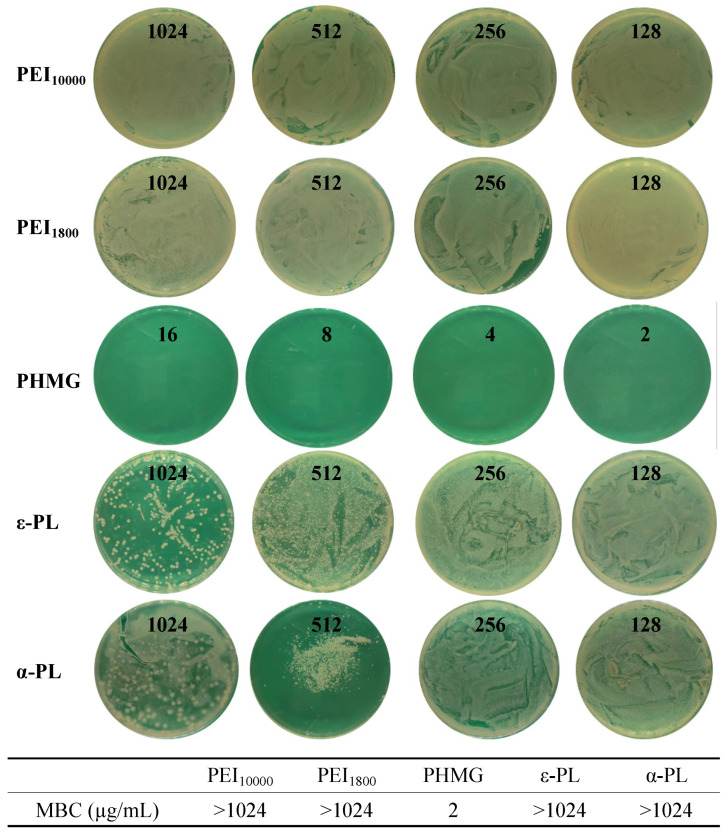
Evaluation of cationic polymers for eliminating persistent MSSA. The unit of polymeric concentration is µg/mL.

The persistent bacteria obtained from the above isolation were treated with the above-described cationic polymers. The results of the bactericidal evaluation experiments (Figure 4) showed that PEI_10000_, PEI_1800_, ε-PL, and α-PL were not bactericidal against persistent *MSSA* up to a concentration of 1024 μg/mL, and only PHMG showed significant antibacterial activity, a result similar to that against ordinary MSSA. Interestingly, PHMG was able to completely eliminate persistent bacteria at a concentration of 2 μg/mL, which was significantly better than that against MSSA. Studies have shown that to withstand antibiotic pressure, persistent bacteria often exhibit reduced membrane potential, decreased membrane fluidity, and increased surface negative charge density, particularly in Gram-positive species [71]. To support this argument, the zeta potentials of MSSA and persistent MSSA were measured (Figure S10). It could be observed that the zeta potential of normal MSSA was –15.7 mV, while that of persister cells was –24.6 mV, which was significantly lower than that of normal MSSA (*p* = 0.0061, **). This elevated negative charge significantly enhances the electrostatic attraction between persisters and the guanidinium groups of PHMG molecules, promoting their efficient accumulation on the bacterial membrane [54]. Consequently, PHMG achieves multi-point binding and membrane insertion through its strong cationic groups, disrupting membrane permeability, inducing leakage of intracellular contents, and ultimately leading to rapid bacterial inactivation. In contrast, metabolically active MSSA maintains a stable membrane structure and lower surface negative charge density, resulting in weaker electrostatic interactions and membrane perturbation by PHMG, and thus lower bactericidal efficiency. As a result, persister cells paradoxically display greater susceptibility to PHMG during their dormant state, enabling their complete eradication at low concentrations.

### 3.6. Antimicrobial Performance of Cationic Polymers Against Persistent MRSA

**Figure 5:**
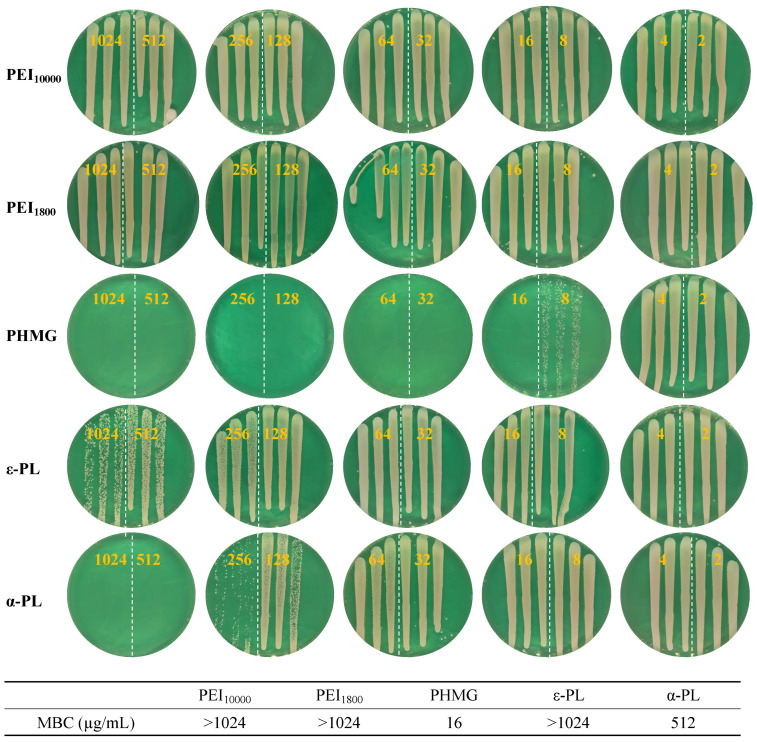
Evaluation of cationic polymers for eliminating persistent MRSA. The unit of polymeric concentration is µg/mL.

Further, we evaluated the bactericidal activity of cationic polymers against persistent MRSA (Figure 5). Similar to the results of the MRSA antimicrobial evaluation, ε-PL and α-PL showed more pronounced bactericidal activity against persistent MRSA compared to PEI_10000_ and PEI_1800_. ε-PL was effective in reducing the number of persistent MRSA colonies at a dose concentration of 1024 μg/mL. α-PL was able to completely remove persistent MRSA at a dose concentration of 512 μg/mL. In the above studies, α-PL generally showed higher antimicrobial activity than ε-PL, possibly due to the correlation between primary amine density and chain length in the polymer. α-PL has a stronger membrane penetration perturbation ability due to its linear structure, higher unit length, and cationic density. PHMG was the most potent against persistent MRSA, completely removing persistent MRSA at a dosage concentration of 16 μg/mL.

In contrast, the bactericidal activity of cationic polymers against persistent MRSA was not significantly enhanced compared to that against regular MRSA cells. This is primarily because both antibiotic-resistant MRSA and its persister subpopulation exhibit similar membrane perturbations and alterations in membrane potential [71]. As a result, the physicochemical properties of their cell membranes remain comparable, unlike the pronounced differences observed between sensitive bacteria and their persister counterparts.

### 3.7. Antimicrobial Performance of Cationic Polymers Against Persistent E. coli

**Figure 6:**
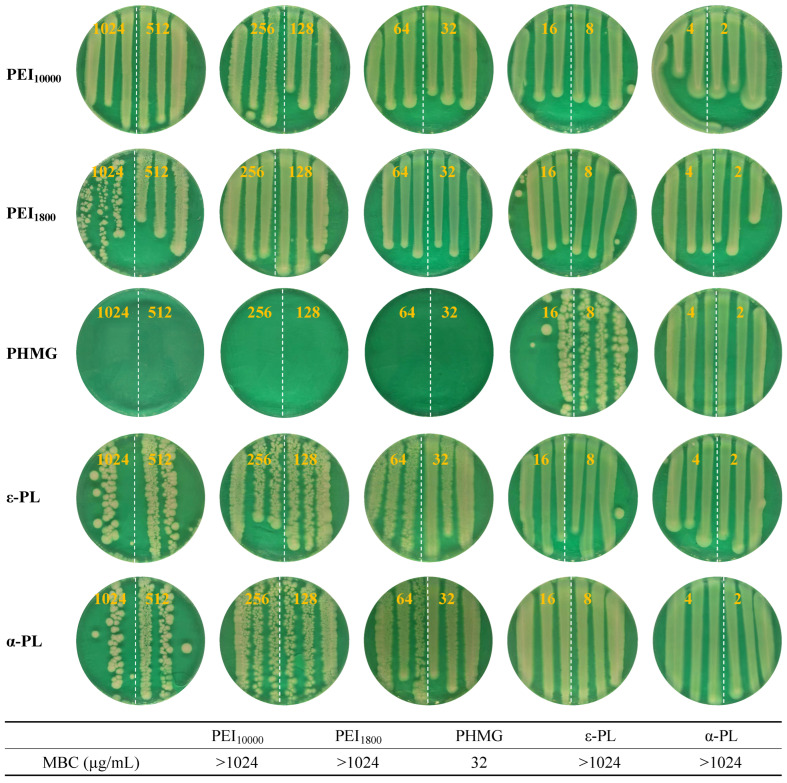
Evaluation of cationic polymers for eliminating persistent *E. coli*. The unit of polymeric concentration is µg/mL.

Evaluation of the bactericidal activity of cationic polymers against persistent *E. coli* (Figure 6) revealed that, among the tested polymers, only PHMG exhibited significant bactericidal effects, completely eradicating the bacteria at 32 μg/mL. In contrast, PEI_10000_ showed negligible antimicrobial activity, with no difference in bacterial growth. Although precise MBC values were not determined for PEI_1800_, ε-PL, and α-PL, noticeable bacterial growth inhibition was observed at the highest tested concentration (1024 μg/mL) compared to the control group, indicating that these three cationic polymers possess modest antimicrobial activity against persistent *E. coli*.

### 3.8. Cytotoxicity Evaluation of Cationic Polymers

The cytotoxicity of five cationic polymers on mouse fibroblasts (L929) was evaluated by the MTT method. Since positively charged cationic materials may cause toxicity to normal cells while destroying bacterial membranes, ensuring the biosafety of cationic polymers is crucial for further applying their antimicrobial properties to clinical applications. Therefore, L929 cells were used to determine the cytotoxicity of five cationic polymers by the MTT method. Gradient concentrations of 1024 μg/mL, 512 μg/mL, 256 μg/mL, 128 μg/mL, 64 μg/mL, 32 μg/mL, 16 μg/mL, and 8 μg/mL were set for experimental investigation.

**Figure 7:**
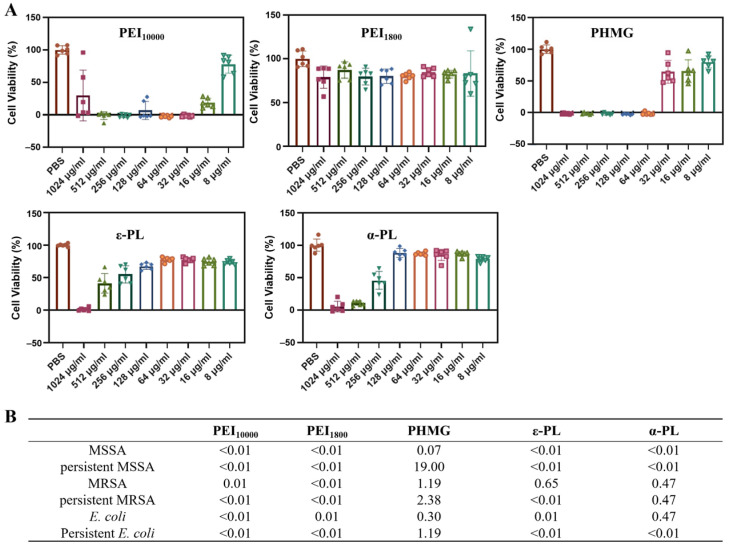
Cytotoxicity evaluation and selectivity index (IC50/MBC) of the cationic polymers. (**A**) Cytotoxicity evaluation. (**B**) Selectivity index (IC50/MBC).

The results (Figure 7A) showed that PHMG exhibited marked cytotoxicity toward L929 cells, with nearly complete cell death observed at concentrations above 32 μg/mL after 24 hours of exposure, while cell viability remained above 50% at concentrations below this threshold. PEI_10000_ demonstrated even higher cytotoxicity, with significant cell death occurring at concentrations above 16 μg/mL and viability exceeding 50% only at concentrations below 8 μg/mL. In contrast, PEI_1800_ displayed minimal cytotoxicity, maintaining cell viability above 50% across all tested concentrations. For ε-PL, concentrations above 1024 μg/mL led to almost complete cell death, whereas viability remained above 50% at concentrations below 256 μg/mL. Similarly, α-PL caused substantial cytotoxicity at concentrations above 512 μg/mL, with cell viability exceeding 50% below 256 μg/mL. Additionally, the selectivity index has been calculated (Figure 7B). The calculations revealed that PHMG demonstrated the strongest selectivity in terms of antibacterial activity, with a particularly notable preference for targeting persisters. Nevertheless, the cytotoxicity of these polymers remains a concern that cannot be overlooked. It is crucial to enhance the activity against persistent bacteria while reducing the biological toxicity of cationic polymers through structural derivation or modification, thereby improving their selectivity index.

## 4. Discussion

In this study, the clearance efficacy of six cationic polymers against sensitive, drug-resistant, and persistent bacteria was systematically evaluated (Table 1). PHMG exhibited the most potent antibacterial activity, with MBCs ranging from 2 to 512 μg/mL against persistent MSSA, MRSA, and *E. coli*, significantly outperforming the other polymers tested. Notably, PHMG completely eradicated persistent MSSA subpopulations at concentrations as low as 2 μg/mL, underscoring its exceptional efficacy against metabolically dormant cells. Natural peptide-based cationic polymers, α-PL and ε-PL, also demonstrated notable activity against persistent MRSA, whereas PEIs exhibited modest antibacterial effects, and PAAm showed no activity. Cytotoxicity assays indicated that PHMG and high-molecular-weight PEI_10000_ induced marked toxicity in mouse fibroblast cells, while α-PL and ε-PL displayed better biocompatibility.

Sensitive, drug-resistant, and persistent bacteria pose infection risks but differ fundamentally in their survival strategies [8,9,14]. Drug-resistant bacteria, such as MRSA, acquire heritable resistance through mutations or horizontal gene transfer, enabling proliferation in antibiotic environments [3]. In contrast, persistent bacteria survive antibiotic exposure through reversible metabolic dormancy, without genetic resistance. Sensitive bacteria remain fully susceptible to antibiotics. These populations also differ in metabolic activity [14]. Sensitive and drug-resistant bacteria remain metabolically active, while persistent bacteria exhibit sharply reduced metabolism. Persistent bacteria present higher surface negative charge density, lower membrane potential, and reduced membrane fluidity [71]. Drug-resistant bacteria like MRSA possess thicker cell walls and greater surface negativity than sensitive strains.

Cationic polymers exert bactericidal effects by targeting bacterial membranes through electrostatic interactions and subsequently leading to membrane disruption. This mechanism bypasses both genetic resistance and metabolic dormancy, contributing to their enhanced efficacy against persistent bacteria. The elevated surface negativity of persistent cells likely strengthens electrostatic interactions [30], while impaired electron transport chain activity may increase membrane susceptibility [71]. However, achieving a balance between antibacterial potency and biocompatibility remains a major challenge, highlighting the need for future structural modifications to optimize the clinical potential of cationic polymers.

## 5. Conclusions

This study systematically evaluated the antimicrobial performance of five typical cationic polymers against sensitive, drug-resistant, and persistent bacteria, clarifying the relationship between polymer structure, cationic group type, and bactericidal efficacy. Results showed that PHMG exhibited the most excellent broad-spectrum bactericidal performance due to its high charge density and strong membrane perturbation ability, especially against MRSA and persistent bacteria, which could be completely eliminated at very low concentrations, significantly outperforming other polymers. Natural peptide cationic polymers, ε-PL and α-PL, also showed good antibacterial activity, with α-PL being more effective than ε-PL in eliminating persistent MRSA. It was confirmed that the elevated negative charge density and reduced membrane stability of drug-resistant and persistent bacteria endow them with higher susceptibility to membrane-acting cationic polymers, providing an effective strategy for combating drug-resistant and persistent infections. Combining antimicrobial performance and biocompatibility results, natural peptide polymers and PHMG show promise for further development. Future work can construct highly efficient and low-toxicity antimicrobial materials and infection prevention and control systems based on their structural properties.

## Figures and Tables

**Table 1:** Summary of bactericidal properties (MBC, µg/mL) of cationic polymers.

	PHMG	PEI_10000_	PEI_1800_	ε-PL	α-PL
MSSA	512	>1024	>1024	>1024	>1024
Persistent MSSA	2	>1024	>1024	>1024	>1024
MRSA	32	1024	>1024	512	512
Persistent MRSA	16	>1024	>1024	>1024	512
*E. coli*	128	>1024	1024	1024	512
Persistent *E. coli*	32	>1024	>1024	>1024	>1024

## Data Availability

The original contributions presented in this study are included in the article/Supplementary Materials. Further inquiries can be directed to the corresponding author.

## Supplementary Materials

The following supporting information can be downloaded at: https://www.mdpi.com/article/10.3390/microorganisms13092002/s1, Figure S1: ^1^H NMR spectrum (400 MHz, D_2_O) of PEI_10000_; Figure S2: ^1^H NMR spectrum (400 MHz, D_2_O) of PHMG.; Figure S3: ^1^H NMR spectrum (400 MHz, D_2_O) of α-PL; Figure S4: ^1^H NMR spectrum (400 MHz, D_2_O) of ε-PL; Figure S5: ^1^H NMR spectrum (400 MHz, D_2_O and DMSO-*d6*) of ε-PL; Figure S6: MIC assay of PAAm against MSSA; Figure S7: MIC assays of chitosan against MSSA; Figure S8: SEM images of MSSA treated with cationic polymers; Figure S9: Number of bacterial colonies of persistent MSSA treated by vancomycin for 24 h; Figure S10: Zeta potential of MSSA and its persistent subpopulation.
