# Supplementary material for: Poly(hexamethylene guanidine): An Effective Compound in Tackling Persistent Bacterial Subpopulations
Source: Microorganisms. 2025 Aug 27;13(9):2002. doi: 10.3390/microorganisms13092002 (PMC12471952; doi:10.3390/microorganisms13092002)
Supplement: Supplementary file 1 [file microorganisms-13-02002-s001.zip › microorganisms-3782080-supplementary.pdf]

Supplementary Information

Poly(hexamethylene guanidine): An Effective Compound in Tackling Persistent Bacterial Subpopulations

Weilin Liu, Jiang Zhang and Liang Chen

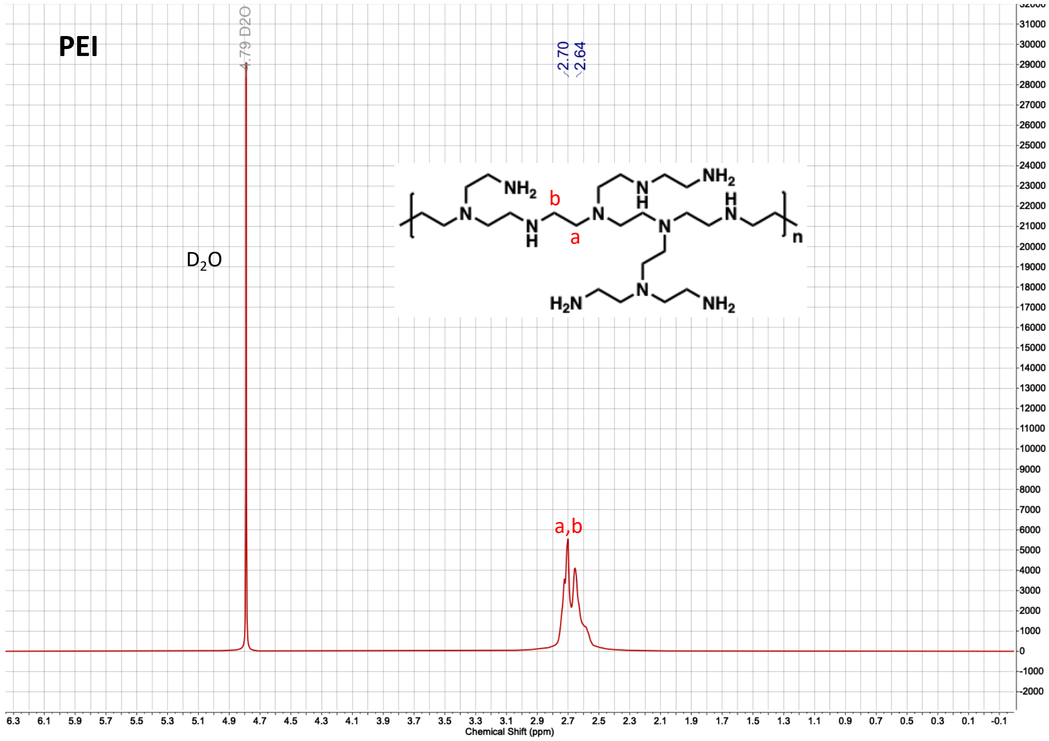

Figure S1. ^{1}H NMR spectrum (400 MHz, D_2O) of PEI_{10000}.

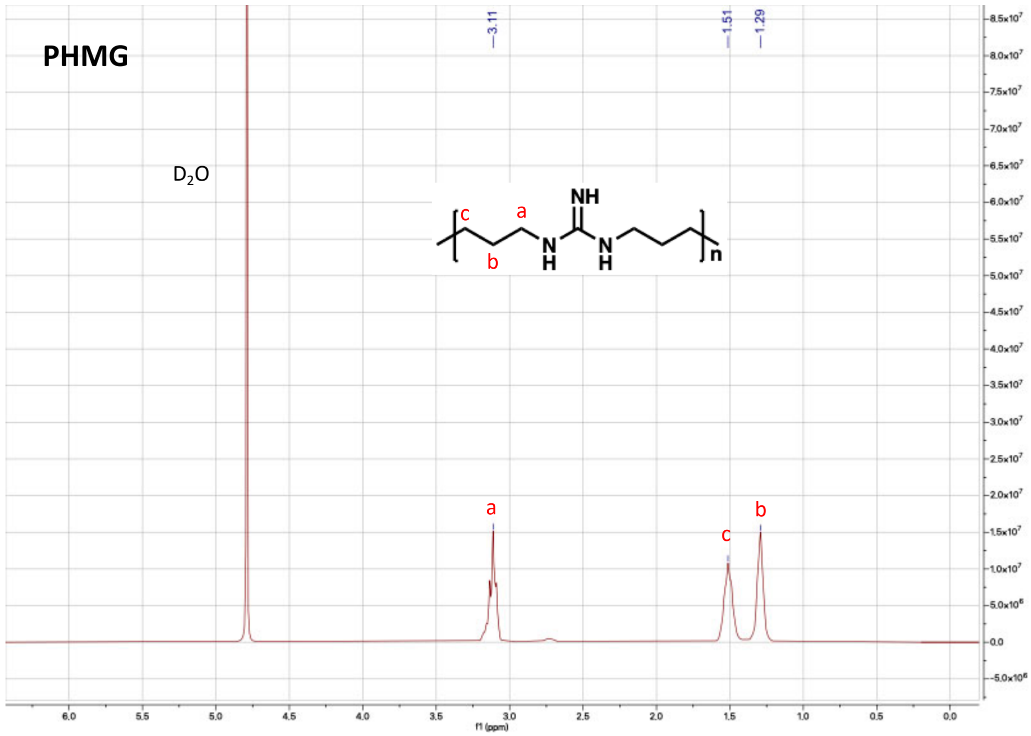

Figure S2. ^{1}H NMR spectrum (400 MHz, D_2O) of PHMG.

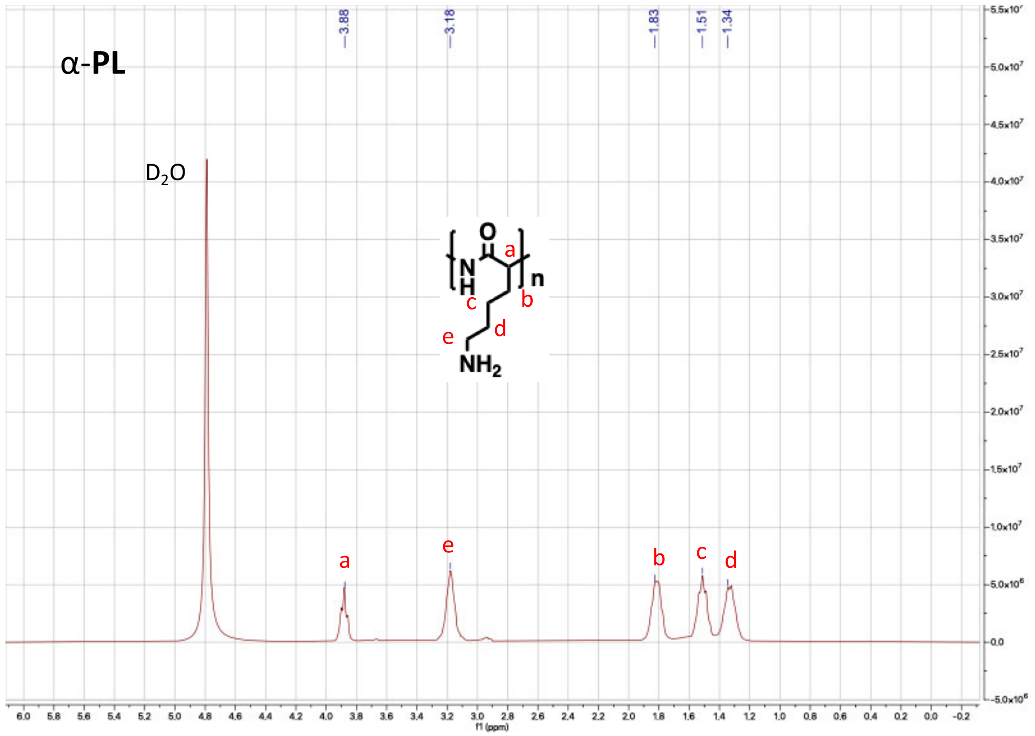

Figure S3. ^{1}H NMR spectrum (400 MHz, D_2O) of α-PL.

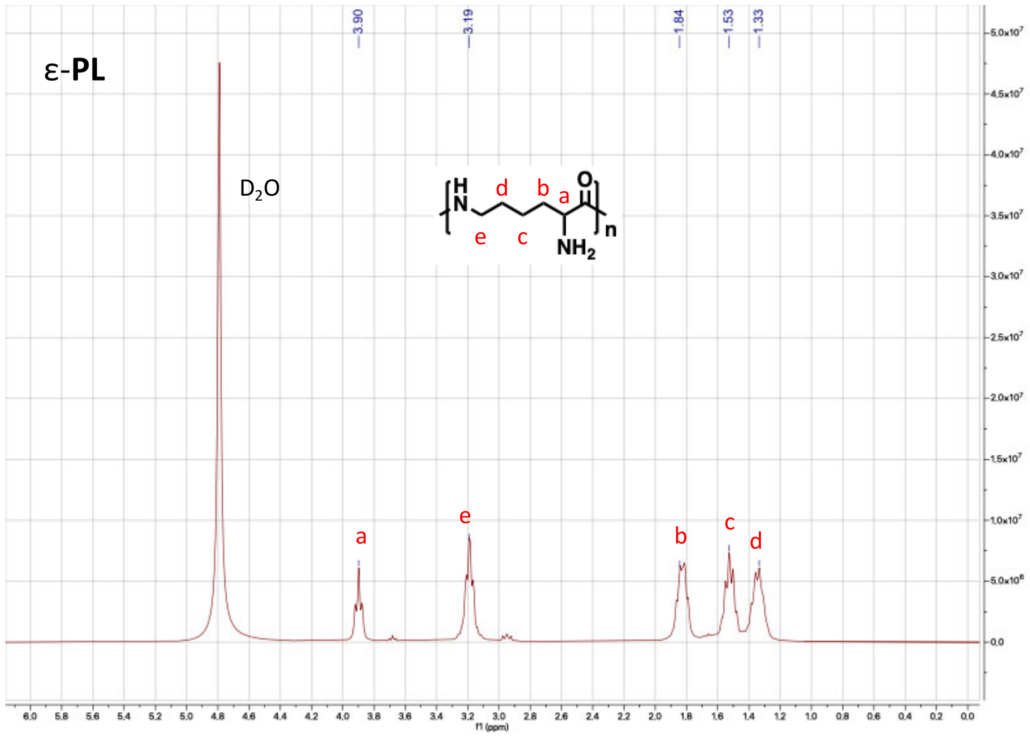

Figure S4. ^{1}H NMR spectrum (400 MHz, D_2O) of ε-PL.

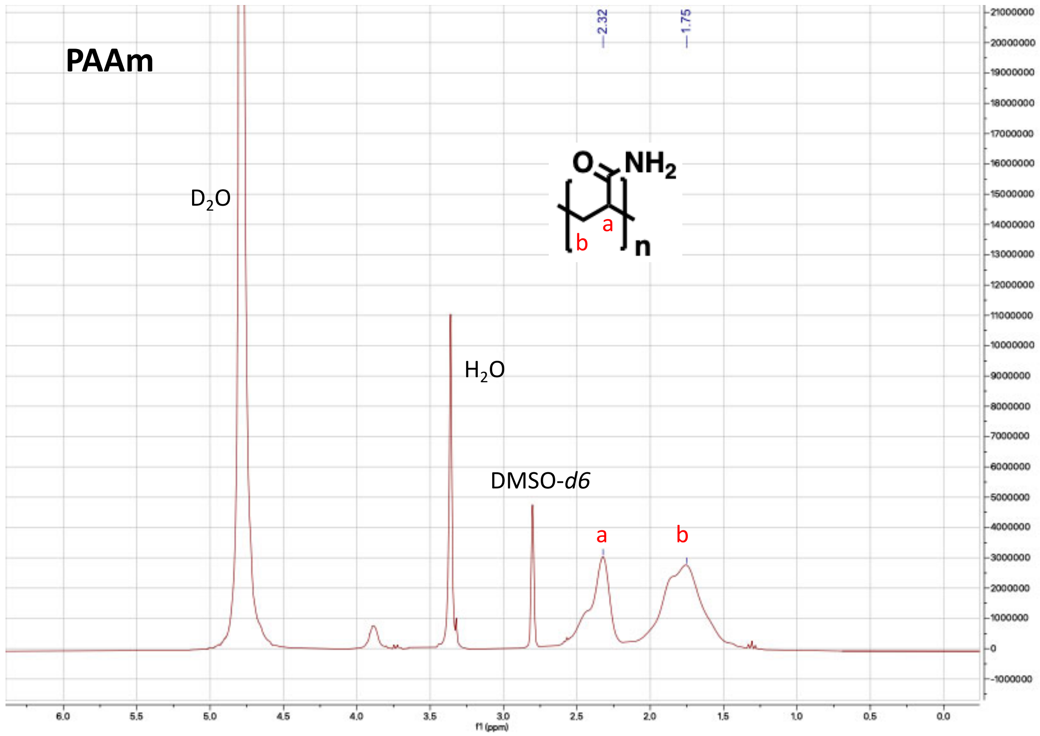

Figure S5. ^{1}H NMR spectrum (400 MHz, D_2O and DMSO-*d6*) of ε-PL.

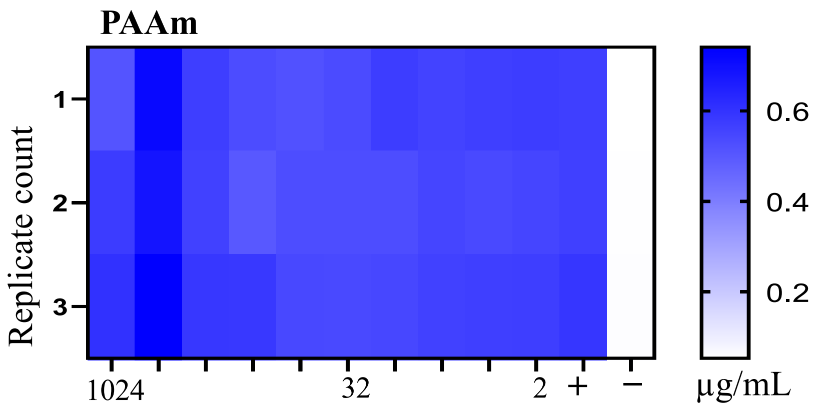

Figure S6. MIC assay of PAAm against MSSA.

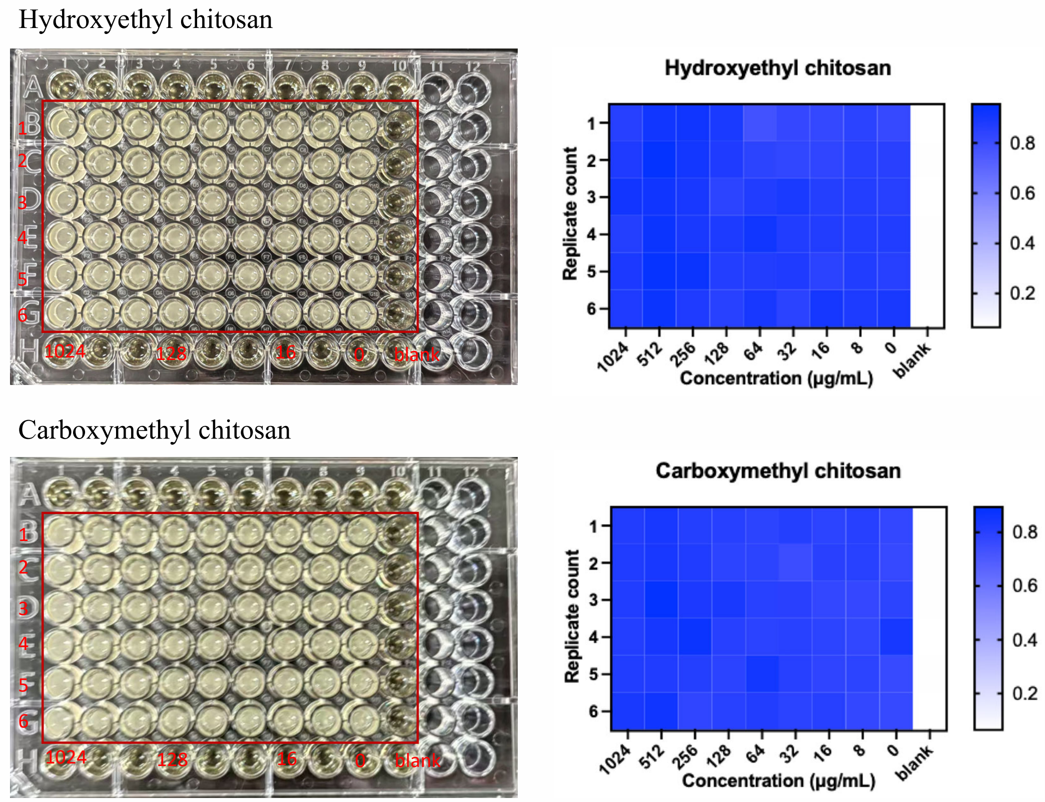

Figure S7. MIC assays of chitosan against MSSA. Hydroxyethyl chitosan (240 KDa, purchased from MACKLIN) and carboxyethyl chitosan (82 KDa, purchased from WAKO) were used against *MSSA* (ATCC 25923).

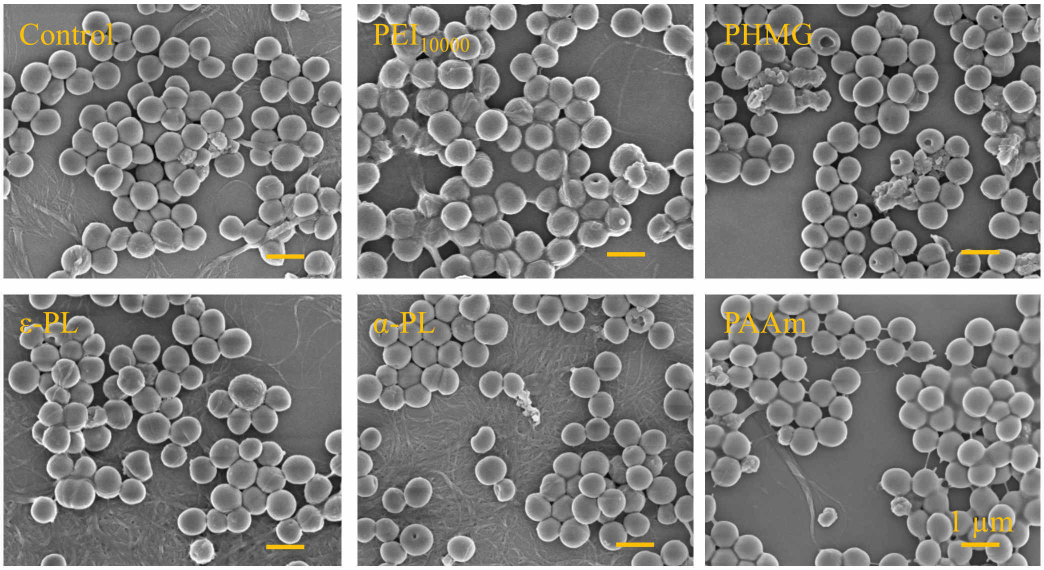

Figure S8. SEM images of MSSA treated with cationic polymers.

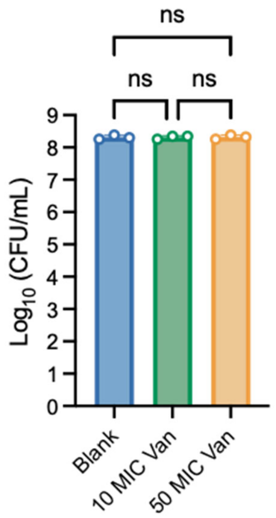

Figure S9. Number of bacterial colonies of persistent MSSA treated by vancomycin for 24 h.

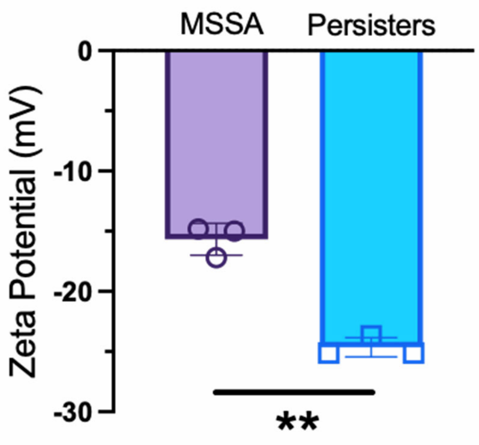

Figure S10. Zeta potential of MSSA and its persistent subpopulation. Bacterial suspensions at a concentration of 1×10^8 CFU/mL were washed three times with 40 mM Hepes buffer and then resuspended in 40 mM Hepes for zeta potential determination.
